# 46,XX testicular disorder of sexual development with *SRY*-negative caused by some unidentified mechanisms: a case report and review of the literature

**DOI:** 10.1186/1471-2490-14-104

**Published:** 2014-12-22

**Authors:** Tian-Fu Li, Qiu-Yue Wu, Cui Zhang, Wei-Wei Li, Qing Zhou, Wei-Jun Jiang, Ying-Xia Cui, Xin-Yi Xia, Yi-Chao Shi

**Affiliations:** Department of Reproduction and Genetics, Institute of Laboratory Medicine, Jinling Hospital, Nanjing University School of Medicine, Nanjing, 210002 PR China; Center for Reproduction and Genetics, Suzhou Municipal Hospital, Nanjing Medical University Affiliated Suzhou Hospital, 26 Daoqian Street, Suzhou, 215002 PR China

**Keywords:** 46,XX testicular disorder of sex development, Ambiguous genitalia, *SRY*-negative

## Abstract

**Background:**

46,XX testicular disorder of sex development is a rare genetic syndrome, characterized by a complete or partial mismatch between genetic sex and phenotypic sex, which results in infertility because of the absence of the azoospermia factor region in the long arm of Y chromosome.

**Case presentation:**

We report a case of a 14-year-old male with microorchidism and mild bilateral gynecomastia who referred to our hospital because of abnormal gender characteristics. The patient was treated for congenital scrotal type hypospadias at the age of 4 years. Semen analysis indicated azoospermia by centrifugation of ejaculate. Levels of follicle-stimulating hormone and luteinizing hormone were elevated, while that of testosterone was low and those of estradiol and prolactin were normal. The results of gonadal biopsy showed hyalinization of the seminiferous tubules, but there was no evidence of spermatogenic cells. Karyotype analysis of the patient confirmed 46,XX karyotype and fluorescent *in situ* hybridization analysis of the sex-determining region Y (*SRY*) gene was negative. Molecular analysis revealed that the *SRY* gene and the AZFa, AZFb and AZFc regions were absent. No mutation was detected in the coding region and exon/intron boundaries of the *RSPO1*, *DAX1*, *SOX9*, *SOX3, SOX10, ROCK1,* and *DMRT* genes, and no copy number variation in the whole genome sequence was found.

**Conclusion:**

This study adds a new case of *SRY*-negative 46,XX testicular disorder of sex development and further verifies the view that the absence of major regions from the Y chromosome leads to an incomplete masculine phenotype, abnormal hormone levels and infertility. To date, the mechanisms for induction of testicular tissue in 46,XX *SRY*-negative patients remain unknown, although other genetic or environmental factors play a significant role in the regulation of sex determination and differentiation.

## Background

46,XX testicular disorder of sex development (DSD) is a rare genetic syndrome, which is characterized by a complete or partial mismatch between genetic sex and phenotypic sex [1]. In addition, 46,XX testicular DSD always presents as one of three phenotypes: (1) classic XX males, infertility with normal male internal and external genitalia; (2) XX males with ambiguous genitalia, which is usually apparent at birth by external genital ambiguities, such as hypospadias, micropenis, or hyperclitoridy, and (3) XX true hermaphrodites with internal or external genital ambiguities detected at birth [2–4]. The sex-determining region Y (*SRY*) gene plays a major role in encoding a testis determining factor (TDF), which is located on the Y chromosome [5, 6]. Approximately 80% of patients with 46,XX testicular DSD are *SRY*-positive and usually have a normal male phenotype at birth. Other *SRY*-negative 46,XX males exhibit different degrees of masculinization [7]. However, even though 46,XX males carry different phenotypes, these patients are often infertile because of the absence of the AZFa, AZFb and AZFc regions [8].

**Table 1:** **Milestones of 46,XX testicular DSD**

Year	Viewpoints		References
1964	46,XX testicular DSD was first described.		Delachapelle *et al*. [1]
1992	*SRY*-positive	The *SRY* gene plays a major role in encoding TDF and indicated that *SRY*-positive 46,XX males were infertile.	McElreavey *et al*. [4]
2001; 2004	*SRY*-negative	Hidden mosaicism was reported to cause TH in some 46,XX *SRY*-negative patients, but the results were differed.	Nieto *et al*. [14]
			Domenice *et al*. [15]
2008; 2011		Up/down regulation of the testis/ovarian signaling pathways was found.	Smith *et al*. [17]
			Tomaselli *et al*. [18]
2010–2013	Described the function of the genes located downstream of the *SRY* gene.	*DAX1*, *SOX9*, *SOX3, SOX10, ROCK1, DMRT*	Mizuno *et al*. [13]
			Sukumaran *et al*. [23]
			Moalem *et al*. [24]
			Sutton *et al*. [25]
			Laronda *et al*. [26]
			Polanco *et al*. [27]

Here, we analyzed the clinical characteristics, chromosomal karyotype, and related genes in a male with 46,XX testicular DSD, who referred to our hospital because of abnormal gender characteristics. The aim of the present study was to investigate the different clinical characteristics in different categories of sex reversed 46,XX individuals and the relationships with variable clinical phenotypes and expression levels of the *SRY, RSPO1*, *DAX1*, *SOX9*, *SOX3, SOX10, ROCK1,* and *DMRT* genes. We also reviewed the literature to identify proposed mechanisms to explain the etiology of *SRY*-negative 46,XX sex reversal (Table 1).

## Case presentation

### Participant and clinical data

A 14-year-old male with microorchidism and mild bilateral gynecomastia was referred to our outpatient clinic because of abnormal gender characteristics. A physical examination included measurement of height, assessment of potential gynecomastia, and inspection of the external sex organs. Bilateral volume was calculated as the sum of the volume of both testes. Semen analysis was assessed according to the guidelines of the World Health Organization. Serum levels of follicle-stimulating hormone (FSH), luteinizing hormone (LH), estradiol (E2), prolactin (PRL), and testosterone (T) were also assessed.

## Methods

### Histopathological examination of gonadal tissue

A gonadal biopsy was performed and specimens were obtained, sliced into 4 μm-thick histological sections, and stained with hematoxylin-eosin (HE) for microscopic analysis [9].

### Immunohistochemical staining of gonadal tissue for inhibin and vimentin

Gonadal tissue from the patient was fixed in 10% buffered formalin solution, embedded in paraffin, and sliced into 3-μm sections for immunostaining with monoclonal antibodies against inhibin and vimentin (HZ817454; dilution, 1:400; Enzyme Chain Biotechnology Co., Ltd., Shanghai, China). The tissue sections were dewaxed and subjected to antigen retrieval (pressure cooking for 1 min, 15 psi, in 0.001 M EDTA, pH 8.0). Immunohistochemical staining of inhibin and vimentin was performed using the EnVision™ + System (K5007; Dako Denmark A/S, Glostrup, Denmark) [9].

### Karyotype analysis of G-banding in lymphocytes and fluorescence in situ hybridization (FISH)

Karyotyping of 100 metaphase lymphocytes from peripheral blood was performed by conventional techniques. The X chromosome, Y chromosome, and *SRY* gene were located using FISH with probes specific for the centromeres of the X and Y chromosomes (item no.: 32-111051CEP X with Spectrum Green and CEP Y with Spectrum Orange; Vysis, Inc., Downers Grove, IL, USA) and *SRY* gene (item no.: 30–190079, *SRY* with Orange; Vysis, Inc.) [10].

### Molecular analysis

Genomic DNA from peripheral blood was extracted using the QIAamp DNA Blood Kit (Qiagen GmbH, Hilden, Germany). Three discrete regions (AZFa, AZFb, and AZFc) located on the long arm of Y chromosome were amplified by multiplex polymerase chain reaction (PCR) using primers specific for the diagnosis of microdeletion of the AZFa, AZFb, and AZFc regions, which included sY84, sY86, sY127, sY134, sY254, sY255, *SRY*, and *ZFX/ZFY*. Then, the *RSPO1*, *DAX1*, *SOX9*, *SOX3, SOX10, ROCK1,* and *DMRT* genes were amplified and sequenced [10]. We also analyzed the presence of SRY sequence in fresh gonadal tissues from this 46,XX male case.

### Single nucleotide polymorphism array

Genomic DNA of the patient was extracted and analyzed using the Cytogenetics Whole-Genome 2.7 M Array (Affymetrix, Inc., Santa Clara, CA, USA), especially for the *SOX9* gene, located between 64000 kb and 72000 kb on chromosome 17q24.2–25.1, according to the manufacturer’s protocols. Briefly, genomic DNA was denatured, neutralized, and then amplified by PCR. The PCR products were then purified, fragmented, and end-labeled with biotin. The fragmented, labeled PCR products were then hybridized overnight to the arrays. The variation in copy number was analyzed using Chromosome Analysis Suite software (v1.2.2; Affymetrix, Inc.) [11].

### Ethics statement

The research adhered to the tenets of the Declaration of Helsinki. The Ethics Committee of Jinling Hospital approved the protocols used in this study. Written informed consent was obtained from the parent of the patient for publication of this case report and any accompanying images. A copy of the written consent is available for review by the Editor of this journal.

## Results

The patient was treated for congenital scrotal type hypospadias at the age of 4 years. On physical evaluation, the patient’s weight was 51 kg (age-appropriate mean weight, 53.37 ± 9.29 kg) and height was 155 cm (age-appropriate mean height, 165.90 ± 7.21 cm). The grade of gynecomastia was IA [12]. Pubic hair was normal, the length of stretched penile was 3 cm and bilateral testicular volume was 2 mL. The results of laboratory analysis were as follows: T = 5.8 nmol/L (normal range, 9.4–37 nmol/L), E2 = 0.142 nmol/L (normal range, 0.129–0.239 nmol/L), FSH = 24.4 IU/L (normal range, 1.5–11.5 IU/L), LH = 15.7 IU/L (normal range, 1.1–8.2 IU/L), and PRL = 174.2 mIU/L (normal range, 89.04–826.8 mIU/L). Semen analysis by centrifugation of the ejaculate indicated azoospermia and no spermatogenic cells were observed. The levels of seminal plasma fructose, α-glucosidase, and acid phosphatase were 2.08 g/L (normal range, 0.87–3.95 g/L), 51.93 U/mL (normal range, 35.1–87.7 U/mL), and 12.37 U (normal range, 48.8–208.6 U), respectively.

**Figure 1:**
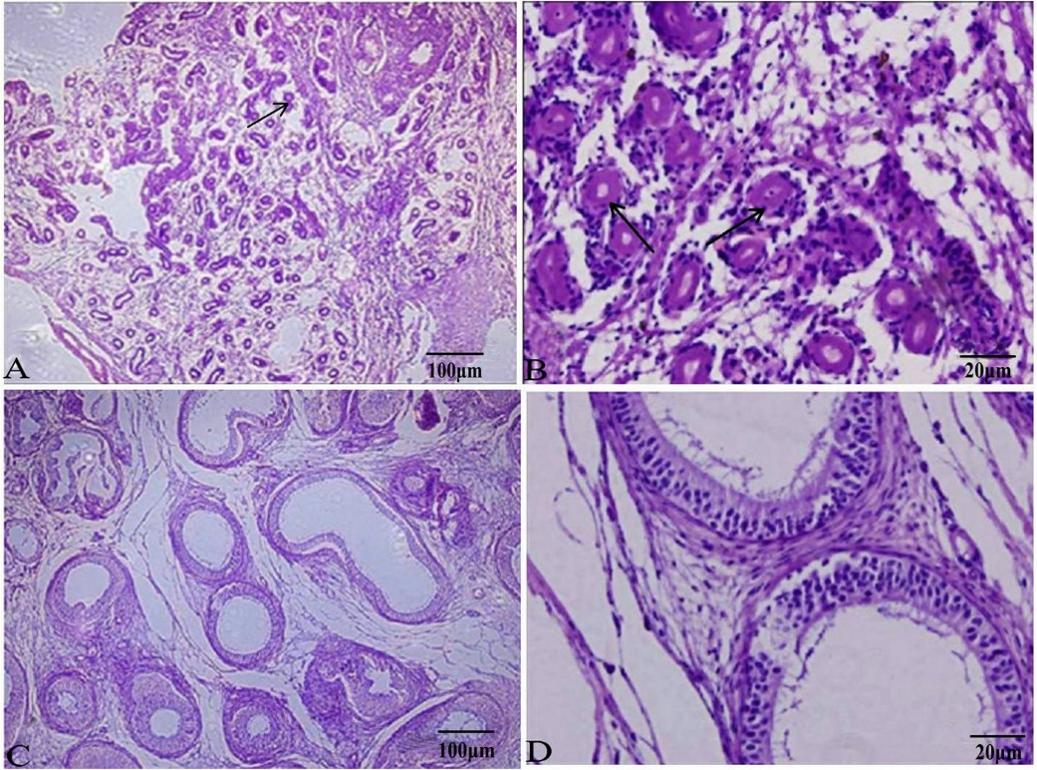
**Histological examination of gonadal tissue by HE staining under light microscopy.** The results of gonadal biopsy showed the appearance of hyalinization of the seminiferous tubules, but without evidence of spermatogenic cells. **(A)** and **(B**) are testis tissue; **(C)** and **(D)** are epididymis tissue. The black arrows show hyalinization of seminiferous tubules in **(A)** and **(B)**.

**Figure 2:**
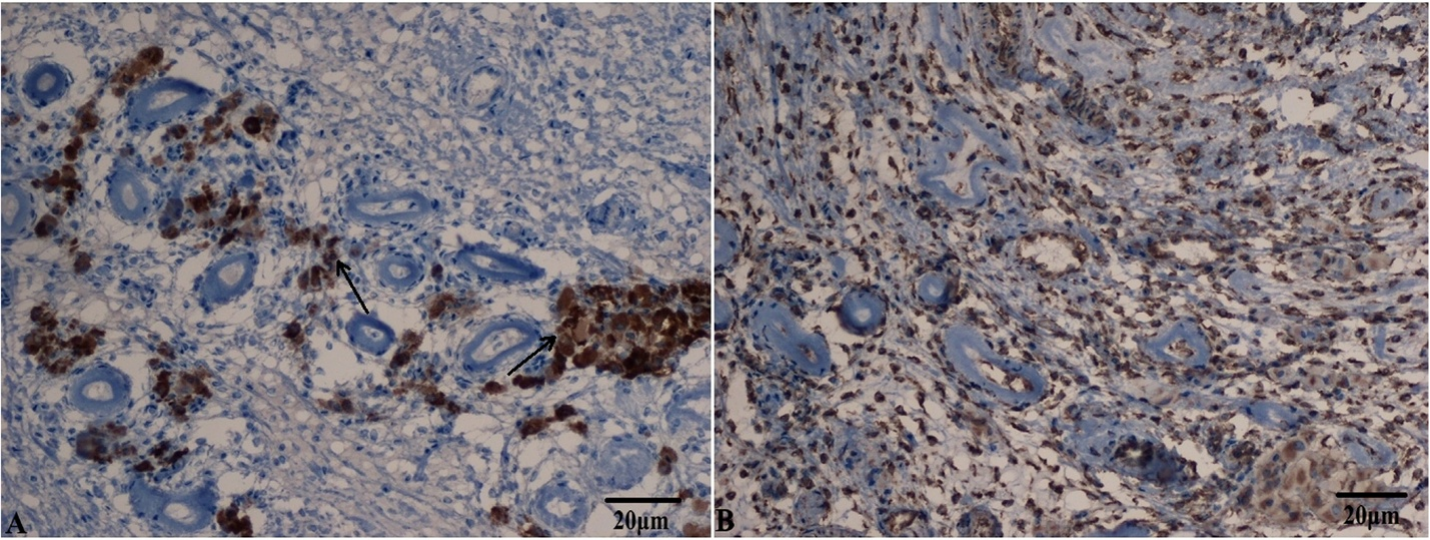
**Immunohistochemical staining of inhibin and vimentin was observed by light microscopy.** Immunohistochemical analysis also confirmed the gonadal tissue had positive immunoreactivity to inhibin **(A)** and vimentin **(B)**, which indicated the presence of Leydig cells. The black arrows in **(A)** indicated that the tissue was positive for inhibin by immunohistochemical staining.

**Figure 3:**
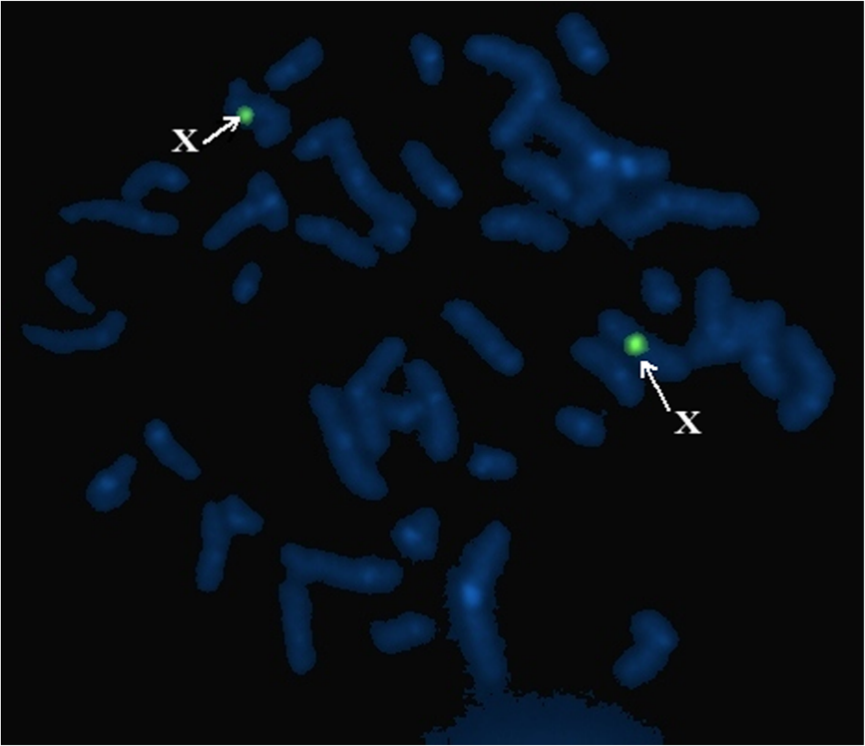
**FISH was used to analysis of metaphase chromosomes of a second case using the LSI** ***SRY*** **(orange) / CEP X (green) probes.** FISH showed the absence of the SRY gene in our patient, while there were two green signals of the X chromosome (white arrows).

**Figure 4:**
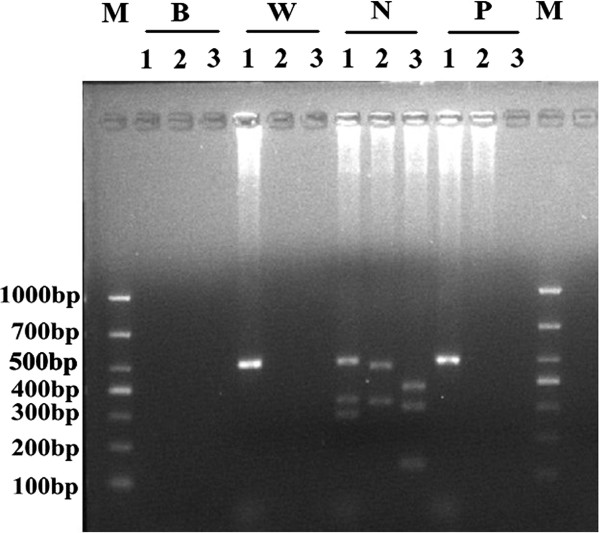
**Result of multiplex polymerase chain reaction (PCR).** Multiplex 1: ZFX/ZFY (690 bp), sY84 (320 bp), and sY127 (274 bp); Multiplex 2: SRY (472 bp) and sY86 (326 bp); Multiplex 3: sY254 (400 bp), sY134 (301 bp), and sY255 (126 bp). M: DL1000 DNA Marker; W: a DNA sample from a woman as a negative control; N: a DNA sample from a normal fertile man as a positive control; P: a DNA sample from the patient; B: a blank (water) control.

**Figure 5:**
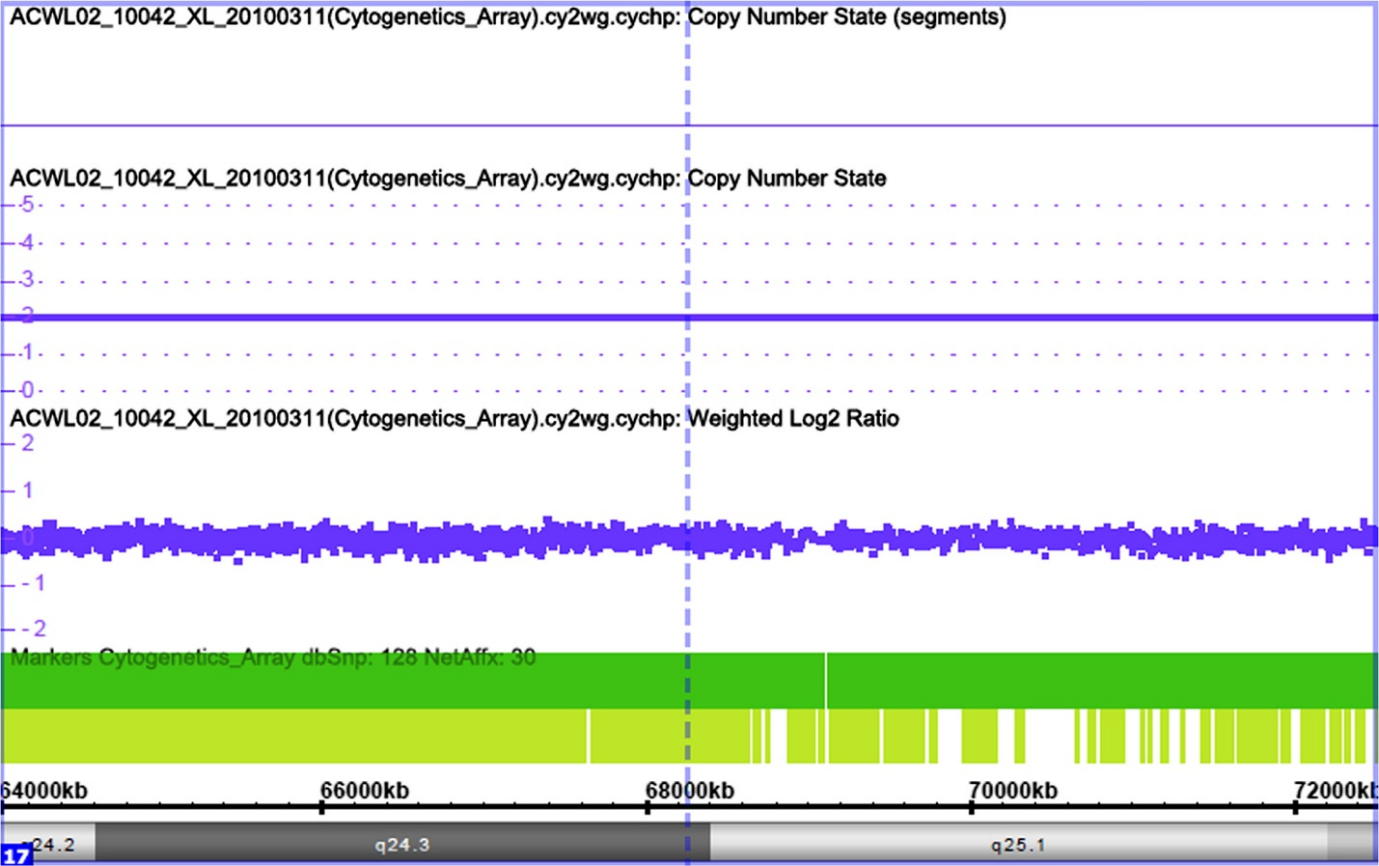
**Affymetrix Cytogenetics Whole Genome 2.7 M Arrays were employed to detect genomic DNA.** No change in the ratio was observed, which indicated no variation in copy number between positions 64000 kb and 72000 kb of chromosome 17q24.2-25.1.

The gonadal biopsy results showed hyalinization of the seminiferous tubules, but without evidence of spermatogenic cells (Figure 1). Consistent with gonadal biopsy, immunohistochemical analysis also confirmed that the gonadal tissue was immunoreactivity positive to inhibin and vimentin, respectively (Figure 2), which indicated the presence of Leydig cells. Karyotype analysis of the patient confirmed a 46,XX karyotype and FISH analysis was negative for the SRY gene (Figure 3). Molecular analysis revealed locus deletions at SY84, SY86, SY127, SY134, SY254, and SY255 within the AZF sequence on chromosome Y, with the absence of the *SRY* gene (Figure 4). Sequencing of the coding regions and exon/intron boundaries of the *RSPO1*, *DAX1*, *SOX9*, *SOX3, SOX10, ROCK1,* and *DMRT* genes detected no mutations. No copy number variation in the whole genome sequence was found. In particular, there was no variation in copy number between positions 64000 kb and 72000 kb of chromosome 17q24.2-25.1. Duplication of this region, which codes for *SOX9*, can trigger sex reversal (Figure 5).

## Discussion

46,XX testicular DSD is a rare form of sex reversal in infertile men, which was first described by la Chapelle *et al.* in 1964 with a frequency of 1:20,000 of newborn males [1]. Most males have normal phenotypes at birth and are usually diagnosed in adolescence because of delayed puberty, gynecomastia, or infertility. Moreover, some XX males have hypospadias, cryptorchidism, or more severe genital ambiguity. However, all 46,XX males are infertile because of the absence of the azoospermia factor region in the long arm of the Y chromosome. 46,XX males can be classified into two subgroups, *SRY*-positive and *SRY*-negative, according to the presence or absence of the *SRY* gene, which is located in the Y chromosome and regulates testicular differentiation [10]. Patients with *SRY*-positive, which always translocates to the X chromosome or to an autosome, are usually more likely to have a normal male phenotype at birth and are referred for infertility treatment after puberty. *SRY*-negative patients include those with ovotesticular-DSD, which is characterized by the presence of testicular and ovarian tissue in the same individual. Testicular-DSD is characterized by full development of both gonads as testes without evidence of ovarian tissue [13].

The mechanisms of testicular tissue induction in *SRY*-negative patients remain unknown, although several hypotheses have been proposed, as follows: (1) hidden gonadal mosaicism for *SRY*; (2) mutations in some autosomal or X-linked genes that repress the male pathway can result in de-repression of the male pathway in XX gonads; and (3) altered expression of other sex determining genes downstream of the *SRY*.

True hermaphroditism (TH), also known as ovotesticular-DSD, is a rare form of intersexuality characterized by the presence of testicular and ovarian tissue in the same individual. Genetic heterogeneity has been proposed as a cause of dual gonadal development in some cases and recently, hidden mosaicism was reported to cause TH in some *SRY*-negative 46,XX patients [14]. However, Domenice *et al*. [15] reported the absence of the SRY sequence in DNA from blood samples of all true hermaphrodites and in testicular and ovarian tissues of a 46,XX true hermaphrodite case and a 46,XX male with ambiguous genitalia, which indicated that cryptic SRY mosaicism in gonadal tissues is not the usual mechanism responsible for testicular development in patients with 46,XX true hermaphroditism.

R-Spondin1 (*RSPO1*) is a novel regulator of ovary development through the up-regulation of the Wnt/β-catenin signaling pathway to oppose testis formation [16, 17]. Loss-of-function mutations in human *RSPO1* lead to reduced β-catenin protein and WNT4 mRNA levels, consistent with downregulation of ovarian pathways, which cause testicular differentiation in 46,XX females [18].

Previous reports have identified gene dysregulation in 46,XX *SRY*-negative DSD. The *SRY*-box 9 (*SOX9*) gene is a widely expressed transcription factor that plays several relevant functions during development and essential for testes differentiation, which is considered to be the direct target gene of the *SRY* [19]. The essential role of *SOX9* in male gonadal development was initially described in mice and subsequent reports stated that *SOX9* duplications were a relevant cause of *SRY*-negative XX sex reversal in dogs [20]. *SOX9* duplication was reported for the first time in Korea in a case involving a 4.2-year-old SRY-negative 46,XX boy with complete sex reversal, which indicated that *SOX9* duplication was a rare cause of 46,XX testicular DSD in humans [21]. The genomic domain regulating SOX9 expression spans more than 1 Mb upstream of SOX9 [11]. Benko *et al*. [22] revealed that a non-coding regulatory region located far upstream of the *SOX9* promoter was critical for gonadal *SOX9* expression and subsequent normal sexual development and copy number variations were the genetic basis of isolated 46,XX testicular DSD of variable severity. Moreover, the minimum critical region associated with gonadal development, about a 67-kb region located 584–517 kb upstream of *SOX9*, was confirmed to induce *SOX9* overexpression in female-to-male sex reversal [11]. Regulatory elements in the duplication region might enhance SOX9 expression through complex mechanisms. It has been suggested that changes in SOX9 expression resulting from disruptions in some regions upstream of SOX9 could be important in 46,XX testicular disorder of sexual development. However, other groups have suggested that duplication in the region of 17q that contains *SOX9* is not a common cause of testis development in subjects with *SRY*-negative 46,XX testicular or ovotesticular DSD [23].

Consistent with *SOX9*, the *DAX1* gene might also function downstream of the *SRY* gene in the sex-determination pathway. Overexpression of the *DAX1* gene could cause female-to-male sex reversal [24]. *ROCK1* (Rho-associated, coiled-coil protein kinase 1) phosphorylates and activates *SOX9* in Sertoli cells to initiate testes formation [13]. *SOX3* was recently shown to upregulate *SOX9* expression via a similar mechanism to *SRY* and modulate XX male sex reversal in humans through gain-of-function mutations mediated by genomic rearrangements around *SOX3*, possibly leading to its altered regulation [25–27]. Overexpression of the *SOX10* gene at 22q13 might be the cause of sex reversal [28].

However, the above mechanisms were not applicable in this case, as any hidden gonadal mosaicism for *SRY* or mutations to autosomal or sex-linked genes that repress the male pathway and alter expression of other sex determining genes downstream of *SRY* were found in our case. These findings suggest that other unidentified genetic or environmental factors play significant roles in the regulation of sex determination and differentiation.

## Conclusions

46,XX *SRY*-negative individuals with complete masculinization are rare and usually exhibit phenotypic differences. Our patient had incomplete masculinization, which was characterized by microorchidism and mild bilateral gynecomastia, and was diagnosed with abnormal gender characteristics. His T level was low and levels of FSH and LH were elevated. Furthermore, we detected locus deletions at SY84, SY86, SY127, SY134, SY254, and SY255 within the AZF sequence on chromosome Y, with the absence of the *SRY* gene. Further studies are needed to explain the mechanisms of 46,XX testicular disorder of sexual development characterized by the described phenotype with a lack of spermatogenesis.

## Consent

Written informed consent was obtained from the parent of the patient for publication of this case report and any accompanying images. A copy of the written consent is available for review by the Editor of this journal.

## Abbreviations

DSD: Disorder of sex development
*SRY*: Sex-determining region Y
TDF: Testis determining factor
FSH: Follicle-stimulating Hormone
LH: Luteinizing Hormone
E2: Estradiol
PRL: Prolactin
T: Testosterone
PCR: Polymerase Chain Reaction
FISH: Fluorescent in situ hybridization
TH: True hermaphroditism
Rspo1: R-Spondin1
*SOX9*: *SRY*-box 9
*ROCK1*: Rho-associated, coiled-coil protein kinase 1.
